# Supplementary material for: Increased influence of prior choices on perceptual decisions in autism
Source: eLife. 2021 Jul 7;10:e61595. doi: 10.7554/eLife.61595 (PMC8289410; doi:10.7554/eLife.61595)
Supplement: Transparent reporting form [file elife-61595-transrepform.docx]

***eLife’s* transparent reporting form**

We encourage authors to provide detailed information *within their submission* to facilitate the interpretation and replication of experiments. Authors can upload supporting documentation to indicate the use of appropriate reporting guidelines for health-related research (see [EQUATOR Network](http://www.equator-network.org/%20)), life science research (see the [BioSharing Information Resource](https://biosharing.org/)), or the [ARRIVE guidelines](http://www.plosbiology.org/article/info:doi/10.1371/journal.pbio.1000412) for reporting work involving animal research. Where applicable, authors should refer to any relevant reporting standards documents in this form.

If you have any questions, please consult our Journal Policies and/or contact us: [editorial@elifesciences.org](mailto:editorial@elifesciences.org).

**Sample-size estimation**

- You should state whether an appropriate sample size was computed when the study was being designed
- You should state the statistical method of sample size computation and any required assumptions
- If no explicit power analysis was used, you should describe how you decided what sample (replicate) size (number) to use

Please outline where this information can be found within the submission (e.g., sections or figure legends), or explain why this information doesn’t apply to your submission:

The sample size for the location discrimination task in this study was determined based on our previous study (Feigin, H. *et al.* Perceptual decisions are biased toward relevant prior choices. *Sci Rep* 11, 648 (2021). https://doi.org/10.1038/s41598-020-80128-0) which tested the same paradigm in young adults. Although performance in children may differ, the task was identical, and therefore this offered the best available estimate. Specifically, that study found significant effects of prior choices (in a variety of conditions) when using sample sizes of around 20. Hence, that served as a benchmark sample size for this study. Power analysis was performed for finding a difference between ASD and control groups in the primary condition of this study (using the effect size from the same condition in the previous study) at α= 0.05 significance level and 1-β = 0.90 power (assuming normal distributions, equal variances and using uncorrected comparisons). This estimated that as few as 7 participants were required (per group) to find a difference in β*_prev_choices_* coefficients and that 22.5 participants were required (per group) to find a difference in ΔPSEs. With the challenges associated with ASD recruitment we aimed for N > 20 participants per group (and attained 23 ASD and 27 control participants). Heading discrimination analysis was done on previously published data (Zaidel et al., 2015) hence power analysis was not performed.

**Replicates**

- You should report how often each experiment was performed
- You should include a definition of biological versus technical replication
- The data obtained should be provided and sufficient information should be provided to indicate the number of independent biological and/or technical replicates
- If you encountered any outliers, you should describe how these were handled
- Criteria for exclusion/inclusion of data should be clearly stated
- High-throughput sequence data should be uploaded before submission, with a private link for reviewers provided (these are available from both GEO and ArrayExpress)

Please outline where this information can be found within the submission (e.g., sections or figure legends), or explain why this information doesn’t apply to your submission:

For the experiment in this study (location discrimination) one group of ASD and one control group were tested (no replicates were tested). The analysis of heading discrimination data were also based on one group of ASD and one control group. The exclusion criteria for handling outliers (according to goodness-of-fit of the psychometric functions) are described in Methods section “Data Analysis”.

**Statistical reporting**

- Statistical analysis methods should be described and justified
- Raw data should be presented in figures whenever informative to do so (typically when N per group is less than 10)
- For each experiment, you should identify the statistical tests used, exact values of N, definitions of center, methods of multiple test correction, and dispersion and precision measures (e.g., mean, median, SD, SEM, confidence intervals; and, for the major substantive results, a measure of effect size (e.g., Pearson's r, Cohen's d)
- Report exact p-values wherever possible alongside the summary statistics and 95% confidence intervals. These should be reported for all key questions and not only when the p-value is less than 0.05.

Please outline where this information can be found within the submission (e.g., sections or figure legends), or explain why this information doesn’t apply to your submission:

The statistical approach is presented in a dedicated section entitled “Statistics” in the Methods. Statistical details (including specific p-values, statistics, 95% confidence intervals, effect size, methods of multiple test correction and the test used) are provided together with the results as they are reported in the Results section.

(For large datasets, or papers with a very large number of statistical tests, you may upload a single table file with tests, Ns, etc., with reference to sections in the manuscript.)

**Group allocation**

- Indicate how samples were allocated into experimental groups (in the case of clinical studies, please specify allocation to treatment method); if randomization was used, please also state if restricted randomization was applied
- Indicate if masking was used during group allocation, data collection and/or data analysis

Please outline where this information can be found within the submission (e.g., sections or figure legends), or explain why this information doesn’t apply to your submission:

Participants were allocated into groups according to their clinical condition – ASD or healthy controls (this was not masked).

**Additional data files (“source data”)**

- We encourage you to upload relevant additional data files, such as numerical data that are represented as a graph in a figure, or as a summary table
- Where provided, these should be in the most useful format, and they can be uploaded as “Source data” files linked to a main figure or table
- Include model definition files including the full list of parameters used
- Include code used for data analysis (e.g., R, MatLab)
- Avoid stating that data files are “available upon request”

Please indicate the figures or tables for which source data files have been provided:

Clinical and demographic data are provided in Tables 1 and 2. The behavioral data gathered for this study, and code, were uploaded to github – as detailed in the manuscript section “Data and code”.
